# Determination of Permittivity of Dielectric Analytes in the Terahertz Frequency Range Using Split Ring Resonator Elements Integrated with On-Chip Waveguide

**DOI:** 10.3390/s20154264

**Published:** 2020-07-30

**Authors:** Sae June Park, John Cunningham

**Affiliations:** School of Electronic and Electrical Engineering, University of Leeds, Woodhouse Lane, Leeds LS2 9JT, UK; s.park@leeds.ac.uk

**Keywords:** permittivity measurement, metamaterials, split-ring resonator, on-chip waveguide

## Abstract

We investigate the use of finite-element simulations as a novel method for determining the dielectric property of target materials in the terahertz (THz) frequency range using split-ring resonator (SRR) sensing elements integrated into a planar Goubau line (PGL) waveguide. Five such SRRs were designed to support resonances at specific target frequencies. The origin of resonance modes was identified by investigating the electric field distribution and surface current modes in each SRR. Red-shifts were found in the resonances upon deposition of overlaid test dielectric layers that saturated for thicknesses above 10 µm. We also confirmed that the SRRs can work as independent sensors by depositing the analyte onto each individually. The relation between the permittivity of the target material and the saturated resonant frequency was obtained in each case, and was used to extract the permittivity of a test dielectric layer at six different frequencies in the range of 200–700 GHz as an example application. Our approach enables the permittivity of small volumes of analytes to be determined at a series of discrete frequencies up to ~1 THz.

## 1. Introduction

Terahertz time-domain spectroscopy (THz-TDS) has been recognized as a powerful tool to detect a range of target materials since it variously provides non-contact [1], label-free [2] and non-destructive [3,4] detection capabilities, each being important across a range of applications. However, the use of THz-TDS to determine dielectric properties is challenging when the volume of the target material is relatively small compared to the wavelength of THz waves, owing to the then low scattering cross section between THz waves and target material, which limits the interaction efficiency [5]. On the other hand, on-chip THz spectroscopies provide a planar geometry onto which unknown dielectric films or analytes may be deposited, thereby exploiting the highly confined THz electric or plasmonic fields compared to free-space THz-TDS geometries [6]. Such systems can thereby offer an ideal platform to investigate the transmission characteristics of target materials when only small amounts of analyte or samples are available, or when the characteristics of small devices are to be interrogated. Previously, therefore, such systems have also been adopted to investigate the THz plasmonic response of individual two-dimensional electron systems [7], and used as dielectric [8] and liquid sensors [9] in the former application. It is therefore pertinent to investigate methods to further reduce the interaction cross section in on-chip THz-TDS systems while improving their detection sensitivity, and thus their range of potential applications.

Metamaterials consist of arrays of resonant elements designed to resonate with incident electromagnetic waves at specific frequencies [10,11,12]. Terahertz metamaterials have received significant attention in the field of biosensors, where they can overcome problems of low-scattering cross section by their highly confined electric fields when on-resonance [13,14,15,16,17], while also affording a degree of tunability [18,19,20,21,22,23]. We recently demonstrated that THz metamaterials can be used to measure the permittivity of thin films and liquids at the resonant frequency of metamaterials using free-space THz-TDS measurements [24]. In order to exploit this method further, the determination of permittivity at multiple frequencies should be carried out across a broadband frequency range. This requires multiple split-ring resonators (SRRs), which can be distinguished from each other by having their resonances at different frequencies.

In general, one requires at least *n* resonators (e.g., split-ring resonators) resonating at *n* fundamental frequencies in order to measure the permittivity of target materials at *n* discrete frequencies. While arrays of SRR elements could be sampled using free-space THz-TDS, sensing locations would then necessarily be dispersed across a chip, so that small analyte volumes could not easily be sampled. On the other hand, on-chip THz systems provide extreme confinement of the THz electric field in an on-chip waveguide, thus potentially allowing the response of both single and small numbers of THz SRRs to be distinguished. We therefore believe that it is desirable to combine SRRs with on-chip THz systems, which allows us to propose a novel method to determine the permittivity of target materials at multiple (THz) frequencies at several spatially proximal but distinct locations, with very small (<~8 femtoliter) volumes of analyte required.

In this work, we present finite-element calculations of the resonant frequency of SRRs integrated with on-chip THz waveguides under conditions of dielectric loading (representing analytes) to extract their dielectric properties. An exemplar system using five SRRs was designed, with these elements integrated into a planar Goubau line (PGL) waveguide; sequential readout of all the SRRs was then obtained by passing a THz pulse through the waveguide.

## 2. Simulation Results and Discussions

**Figure 1:**
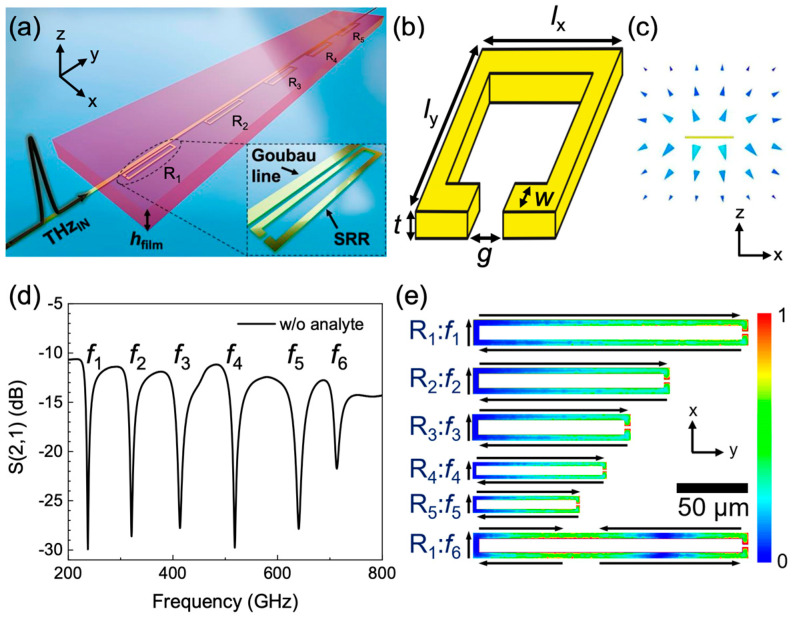
(**a**) Schematic of the simulation model used. Five split-ring resonators (SRRs) (R_1_–R_5_) are located next to the planar Goubau line (PGL) in the *x*–*y* plane, and the dielectric film thickness is labelled *h*_film_. Inset shows the SRR R_1_ and the PGL. (**b**) An example of SRR pattern used in the simulations. The geometrical factors such as *l*_x_, *l*_y_, *w* and *g* change in each SRR. Detailed geometrical parameters of the SRRs are listed in Table 1. (**c**) Cross-sectional view of the instantaneous electric field in a cross section of space surrounding the PGL at 500 GHz. (**d**) The transmission parameter S_21_ calculated for the PGL with the integrated SRR device without dielectric loading. Six resonances are labelled as *f*_1_–*f*_6_. (**e**) A colour scale of field magnitude (plotted in arbitrary units) and the surface current direction in the SRRs at resonant frequencies in the *x*–*y* plane at *z* = 0 without dielectric loading.

Split-ring resonators integrated into PGL waveguides were simulated using ANSYS High-Frequency Structure Simulator (HFSS) to calculate the frequency-dependent transmission and thereby the resonant frequencies. SRRs were chosen owing to their geometrical simplicity, making it easy to tune the resonant frequency. Figure 1a shows a schematic of the linear series of SRRs (labelled as R_1_–R_5_) undergoing THz transmission coupled to a 5-μm-wide and 1-mm-long centre-line PGL waveguide, with a dielectric analyte layer loaded on top. The SRRs were each separated from the PGL by 1.5 μm, which was chosen by consideration of a typical resolution limit easily achievable using photolithography. Figure 1b shows an SRR consisting of a rectangle with outer dimensions of *l*_x_ × *l*_y_ and a gap structure of size *g*. The width and the thickness of the metal strip are *w* and *t* respectively. The geometrical parameters of five resonators (R_1_–R_5_) are shown in Table 1.

In order to obtain the transmission spectra (S_21_) of the waveguide with five integrated SRRs, two-port S-parameter simulations were performed. The THz signals were generated from wave-ports directly coupled to the PGL, as previously demonstrated in experiments [9,25]. We note that, using this method, the PGL mode is formed without a coplanar waveguide to PGL transition [26]. We also note that the electric field excitation at the wave port showed a quasi-transverse electromagnetic mode, which was coupled by the PGL. In order to demonstrate further that this method produces a PGL mode propagating along the transmission line, a cross-sectional view of the electric field was plotted at 500 GHz using vector representation, as shown in Figure 1c. A radiating instantaneous electric field pattern distributed around the centre conductor was found, with all electric field vectors pointing away from the PGL, and perpendicular to the waveguide, indicating a pure Goubau mode. We note that (i) since we used S_21_ to determine the effect of loading the SRRs, our results are robust against any roll-off of pulsed signal caused by limited bandwidth of photoconductive switches or waveguide losses in a real device, and that (ii) a similar approach was previously shown to give good agreement with experimental results on loaded band-stop filters attached to microstrip line [27]. A radiation boundary condition was used in the model to remove any incident electric field on the limits of the simulations. A permittivity of 3.8 was used for the quartz substrate, valid in the frequency range of 50–1100 GHz as previously shown by experiment [28]. The transmission characteristics of the SRRs coupled to the PGL were first examined. Figure 1d shows the simulated transmission spectra of five SRRs integrated in PGL before the deposition of any dielectric analyte. Six resonances were observed at 237, 321, 413, 518, 640 and 713 GHz, labelled as *f*_1_–*f*_6_ in Figure 1d. To identify the origin of these resonances, the electric field distribution and surface current mode of each SRR at resonant frequency were plotted in Figure 1e. R_1_–R_5_ show a highly confined electric field in the gap structure and circulating surface current mode at *f*_1_–*f*_5_ respectively, indicating that *f*_1_–*f*_5_ are fundamental inductive-capacitive (LC) resonant frequencies, while R_1_ also supports a dipole mode at *f*_6_ [29].

**Figure 2:**
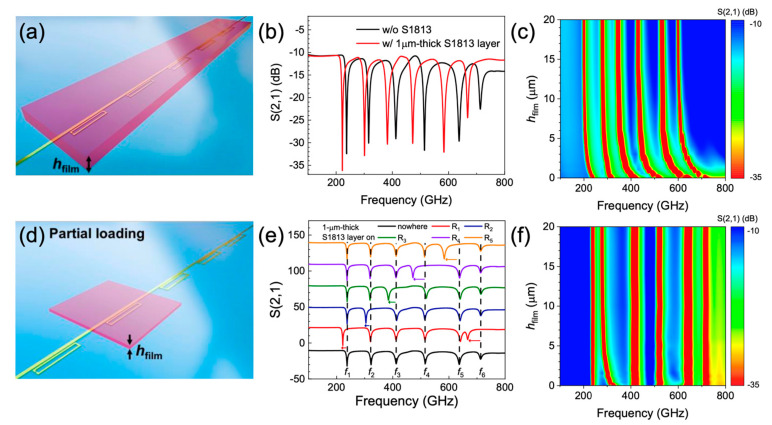
(**a**) Schematic of the SRRs undergoing THz transmission with the loading of S1813 layer on R_1_–R_5_. (**b**) Transmission parameter S_21_ calculated for the SRRs-integrated PGL waveguide with (red line) and without (black line) 1-µm-thick S1813 layer. (**c**) A colour scale plot of S_21_ parameter as a function of *h*_film_. (**d**) Schematic of the SRRs undergoing THz transmission with partial loading of S1813 layer on R_2_. (**e**) A series of the S_21_ parameter when 1-μm-thick S1813 layer was partially deposited on nowhere (black line), R_1_ (red line), R_2_ (blue line), R_3_ (green line), R_4_ (violet line), and R_5_ (orange line). (**f**) A colour scale plot of the S_21_ parameter as a function of *h*_film_ with partial loading of S1813 layer on R_2_.

To study the effect of dielectric loading on the resonant frequencies, we investigated the transmission characteristics for varying thicknesses (*h*_film_) of dielectric layers deposited onto the system, as shown in Figure 2a. We chose a flat permittivity of ~2.97 for the common dielectric S1813 (positive photoresist, Shipley Inc.) since this has been well-characterised across the THz frequency range [30]. Figure 2b shows the simulated THz transmission of the SRRs with and without the deposition of an S1813 layer with a thickness of 1 μm (*h*_film_ = 1 μm). Red-shifts were found in the resonant frequencies as a 1-μm-thick S1813 layer was loaded, owing to an increase in the effective permittivity near the SRRs [31,32]. Here, the reference signal is the transmission parameter S_21_ calculated for a SRRs-integrated PGL device on a quartz substrate without a dielectric overlayer. The size of the resulting frequency shifts were found to be 15, 15, 30, 42, 54 and 45 GHz for *f*_1_–*f*_6_, respectively. The resonant frequency (*f*) upon deposition of the dielectric film can be expressed by *f* = *f*_0_(ε/ε_eff_)^−1/2^, where *f*_0_ is the resonant frequency without the dielectric film, and ε and ε_eff_ are the effective permittivities near the SRRs with and without the dielectric loading, respectively [24]. Here, the relation between ε and ε_eff_ can be expressed as ε = ε_eff_ + α(ε_film_−ε_air_), where ε_film_ and ε_air_ are the permittivities of the dielectric film and the air, respectively, and α is the sensitivity coefficient, which is determined by the cross section of interaction between the electric field pattern above the device and the overlaid dielectric [24].

Figure 2c shows a colour scale plot of the S_21_ transmission parameter of the SRRs as a function of *h*_film_. The resonant frequencies decrease as the S1813 thickness increases with the refractive index unit (RIU) surface sensitivity (Δ*f*/(*h*_film_·(ε_film_^1/2^ − 1))) of 21, 21, 42, 58, 75 and 63 GHz/µm for *f*_1_–*f*_6_, respectively when *h*_film_ < 1 μm, but then saturates at a specific film thickness (*h*_sat_). This implies that a limited detection volume can be ascribed to the electric field distribution near the SRRs at the resonant frequency, owing to the effective confinement of electric field near the structure [20,30,33]. The relation between *f* and the saturated resonant frequency (*f*_sat_) can be expressed as Δ*f* = Δ*f*_sat_(1 − exp(–*h*_film_/*h*_sat_)), where Δ*f* = *f*_0_ − *f*, and Δ*f*_sat_ = *f*_0_ − *f*_sat_ [24]. We extracted *h*_sat_ of 2.4, 3, 2.4, 1.7, 1.5 and 2.3 µm for *f*_1_–*f*_6_, respectively. At the same time, Δ*f*_sat_ were found to be 39, 39, 71, 84, 106 and 117 GHz for *f*_1_–*f*_6_, respectively, corresponding to an RIU sensitivity (Δ*f*/(ε_film_^1/2^ − 1)) of 54, 54, 99, 117, 147 and 163 GHz. We note that Δ*f*_sat_ is determined by ε_film_ once the saturation condition (*h*_film_ > *h*_sat_) is attained. 

Figure 2d is a schematic of the SRRs undergoing THz transmission with a partial loading of the S1813 layer, showing how each SRR works as a spatially independent dielectric sensor. Figure 2e shows a series for the S_21_ parameter when a 1-μm-thick S1813 layer was partially deposited onto R_1_–R_5_. We note that each SRR in our model works independently since only the resonances from the SRR covered by the S1813 layer were shifted. As an example, Figure 2f shows a colour scale plot of the S_21_ parameter of SRRs as a function of *h*_film_ when S1813 was deposited only on R_2_. It is clear that only *f*_2_ shifts while other resonances remain at the same frequency. We note that the other SRRs also show similar behaviour upon partial dielectric loading for varying thickness.

**Figure 3:**
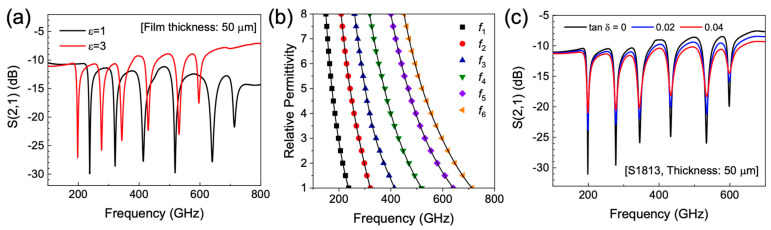
(**a**) Transmission parameter S_21_ calculated for the SRRs-integrated PGL device with a 50-µm-thick overlaid dielectric layer for ε_film_ = 1 and ε_film_ = 3 (assuming tan δ = 0). (**b**) Resonant frequencies as a function of ε_film_ at h_film_ = 50 μm. (**c**) Transmission parameter S_21_ calculated for the SRRs-integrated PGL device with a 50-µm-thick overlaid S1813 layer for tan δ = 0, 0.02, and 0.04.

As mentioned above, *f*_sat_ is determined by ε_film_ when *h*_film_ ≫ *h*_sat_, so this property can be used to determine the permittivity of unknown target materials at multiple frequencies using the SRRs-integrated PGL device. Figure 3a shows the simulated S_21_ parameter of the SRRs with the deposition of the dielectric film (*h*_film_ = 50 μm) for ε_film_ = 1 and ε_film_ = 3. Here, we chose a film thickness of 50 μm, which is well into the saturated regime (*h*_sat_ ~ 2.5 μm). Again, red-shifts were found due to the increase of the effective permittivity near the SRRs. We note that the resonant frequencies we obtained here are *f*_sat_. Figure 3b shows the saturated resonant frequencies as a function of ε_film_ at *h*_film_ = 50 μm. The obtained relations between *f*_sat_ and ε_film_ for *f*_1_–*f*_6_ are shown as solid lines, and were also fitted using the following equation: ε_film_ = A + B·*f*_sat_ + C·*f*_sat_^2^ + D·*f*_sat_^3^ + E·*f*_sat_^4^. We have tabulated the obtained parameters A, B, C, D and E for *f*_1_–*f*_6_ in Table 2. We note that these relations always work once the saturation condition is attained (*h*_film_ ≫ *h*_sat_), enabling the determination of the permittivity of target materials for small (<~8 femtoliter) quantities of analyte. We note, however, that overlap of fundamental resonances can occur after dielectric loading for permittivities greater than ~3.5. Therefore, the number of resonances present should be identified alongside the frequency shifts in order to unambiguously identify permittivities from the saturated resonant frequencies. We also note that our method is only applicable to materials that have no absorption dips across the simulated frequency range. In Figure 3c, we present the effect of the imaginary part of *ε*_film_ on the resonant frequency shift; we simulated the S_21_ parameter of the SRRs with the deposition of the 50-μm-thick S1813 layer for three different loss tangent values (tan δ) of 0, 0.02 and 0.04. It is clear that the resonance depth decreases as loss tangent value increases, though the resonant frequency shifts are unaffected, indicating that our approach can also be used for lossy dielectrics.

**Figure 4:**
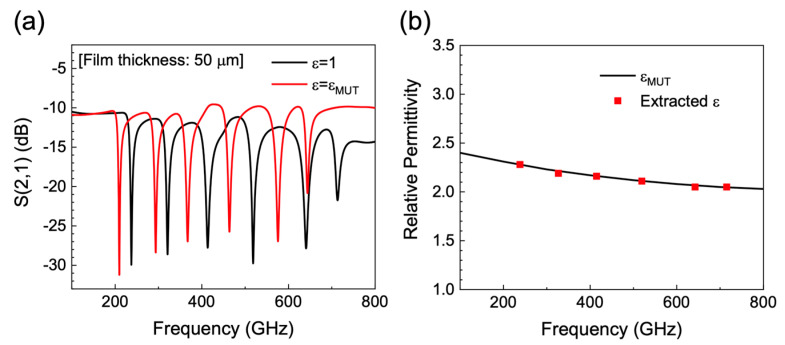
(**a**) Transmission parameter S_21_ calculated for the SRRs-integrated PGL device with a 50-µm-thick overlaid dielectric layer for ε_film_ = 1 and ε_film_ = ε_MUT_. (**b**) Permittivity obtained from *f*_sat_ of SRRs (red boxes) and from the permittivity assigned to the test target material in our simulation model (black line).

To validate our approach, we compared the permittivity of a test target material (ε_MUT_) extracted using our method to the permittivity value assigned to the target materials in HFSS. Figure 4a shows the simulated S_21_ parameter of the SRRs with the deposition of the dielectric film (*h*_film_ = 50 μm) for ε_film_ = 1 and ε_film_ = ε_MUT_. Resonant frequency shifts of 28, 33, 48, 56, 67 and 72 GHz were observed for *f*_1_–*f*_6_, respectively. Using the relations obtained in Figure 3b, the permittivity values of the test target material at six different frequencies were extracted. The extracted permittivity values at six different frequencies (red boxes) using SRRs-integrated PGL are shown together with the permittivity assigned to the test target material in HFSS (black solid line). We extracted ε_film_ of 2.28, 2.19, 2.16, 2.11, 2.05 and 2.05 at fs of 237, 321, 413, 518, 640 and 713 GHz, respectively, in very good agreement with ε_MUT_ with an average error of 0.4%. These results validate our approach, in which the permittivities of various target materials can be determined effectively, without a large volume of analyte or knowledge of the specific film thickness, provided that *h*_film_ ≫ *h*_sat_.

## 3. Conclusions

We proposed a novel approach to determine the permittivity of dielectric target materials at a series of discrete frequencies in the low-THz range (200–700 GHz) using the concept of an array of SRRs integrated into a PGL waveguide. The origin of the resonance modes was identified by the electric field distribution and surface current mode in each SRR. The resonance frequency shift of the SRRs exhibited saturation behaviour with increasing dielectric thickness. *f*_sat_ was determined by ε_film_ when the saturation condition was fulfilled (*h*_film_ ≫ *h*_sat_). The relation between the permittivity of the target material and the saturated resonant frequency was obtained and was used to extract the permittivity of a test dielectric layer at six different frequencies in the range of 200–700 GHz. The extracted permittivity at six different frequencies was in good agreement with the permittivity assigned to the test target material used in our simulation model. Our approach should prove useful for determining the permittivities of various materials at multiple frequencies, without necessitating a large quantity of analyte (<~8 femtoliter per SRR). This work will contribute to the quantitative study of dielectric materials in the THz frequency range.

## Figures and Tables

**Table 1:** Geometrical parameters of split-ring resonators (SRRs) used in this work.

SRR	*f*	*l*_x_ (µm)	*l*_y_ (µm)	*w* (µm)	*g* (µm)	*t* (nm)
R_1_	*f*_1_, *f*_6_	18	194	4	2	150
R_2_	*f*_2_	18	136	4	2	150
R_3_	*f*_3_	18	106.7	4	2	150
R_4_	*f*_4_	11	87	2.4	1.2	150
R_5_	*f*_5_	10.8	69.8	2.4	1.2	150

**Table 2:** Fitting parameters obtained from the relations between *f*_sat_ and ε_film_.

Resonance	A	B	C	D	E
*f*_1_	1.62 × 10^2^	−2.68	1.77 × 10^−2^	−5.41 × 10^−5^	6.32 × 10^−8^
*f*_2_	1.41 × 10^2^	−1.64	7.71 × 10^−3^	−1.68 × 10^−5^	1.39 × 10^−8^
*f*_3_	−4.76	5.03 × 10^−1^	−3.44 × 10^−3^	8.29 × 10^−6^	−6.86 × 10^−9^
*f*_4_	6.50 × 10^1^	−3.53 × 10^−1^	7.58 × 10^−4^	−7.61 × 10^−7^	2.93 × 10^−10^
*f*_5_	8.88 × 10^1^	−4.46 × 10^−1^	9.06 × 10^−4^	−8.71 × 10^−7^	3.27 × 10^−10^
*f*_6_	1.76 × 10^2^	−9.94 × 10^−1^	2.23E × 10^−3^	−2.31 × 10^−6^	9.09 × 10^−10^
